# Expanding D&I science capacity and activity within NIH Clinical and Translational Science Award (CTSA) Programs: guidance and successful models from national leaders

**DOI:** 10.1186/1748-5908-10-S1-A38

**Published:** 2015-08-20

**Authors:** Brian S Mittman, Bryan J Weiner, Enola K Proctor, Margaret A Handley

**Affiliations:** 1 Center for Implementation Practice and Research Support, US Department of Veterans Affairs Greater Los Angeles Healthcare System, Los Angeles, CA, 91343, USA; 2 Department of Research and Evaluation, Kaiser Permanente Southern California, Pasadena, CA, 91101, USA; 3 Department of Health Policy and Management, Gillings School of Global Public Health, University of North Carolina at Chapel Hill, Chapel Hill, NC, 27599, USA; 4 Brown School of Social Work, Washington University in St Louis, St Louis, MO, 63130, USA; 5 Department of Epidemiology & Biostatistics and Department of Medicine, University of California San Francisco, San Francisco, CA, 94110, USA; 6 Center for Vulnerable Populations, San Francisco General Hospital, San Francisco, CA, 94110, USA

## 

The NIH CTSA program (Clinical and Translational Science Awards) supports over 60 research centers working to accelerate scientific discovery, development and implementation to improve health. The CTSA initiative was launched in 2006 to accelerate progress along the research pipeline by overcoming the "translational roadblocks" identified by the Institute of Medicine's Clinical Research Roundtable (CRR)-and discussed in numerous additional reports published in the U.S. and internationally. Although the CRR and its international counterparts identified two broad categories of roadblocks, the first impeding the translation of basic science discoveries into effective clinical and public health interventions and the second impeding appropriate implementation-in-practice and societal benefits of these interventions, to date most CTSA activity has focused largely on the first translational roadblock. The presentations included in this panel represent national leaders in the establishment of effective programs to support and expand CTSA activity related to the second translational roadblock, barriers to effective dissemination, implementation and achievement of population health benefit of NIH-funded research. The presentations describe the components, activities and impacts of CTSA-based D&I science programs established at the University of California at San Francisco, University of North Carolina at Chapel Hill, and Washington University at St. Louis. The panel will also describe the ongoing development of a new program at the University of California at Los Angeles (guided by the three programs featured in the panel) and will feature comments by key national research leaders addressing the value and role of these national leaders in stimulating and guiding similar efforts across other CTSAs and similar research institutions.

## Implications for D&I science

The NIH CTSA program represents a significant investment of research resources in the nation's leading universities and health research institutes. The mission of the program cannot be achieved without a significant increase in efforts to implement CTSA-generated findings and innovations, however, and, hence, without a robust program of implementation research to guide implementation policy and practice. This panel will contribute to ongoing efforts to stimulate and support expanded implementation science interest and activity.

